# Identifying Proteins and Amino Acids Associated with Liver Cancer Risk: A Study Utilizing Mendelian Randomization and Bulk RNA Sequencing Analysis

**DOI:** 10.3390/jpm14030262

**Published:** 2024-02-28

**Authors:** Chi Ma, Ling Tang, Jiaqi Yao, Guang Tan

**Affiliations:** 1 Department of General Surgery, First Affiliated Hospital of Dalian Medical University, Dalian 116011, China; 2 Department of Biochemistry and Molecular Biology, Dalian Medical University, Dalian 116044, China; 3 Department of Anesthesiology, First Affiliated Hospital of Dalian Medical University, Dalian 116011, China

**Keywords:** liver cancer, mendelian randomization, risk, protein, amino acid

## Abstract

Background: Primary liver cancer (PLC) ranks third in terms of fatality rate among all malignant tumors worldwide. Proteomics and metabolomics have become widely utilized in identifying causes and diagnostic indicators of PLC. Nevertheless, in studies aiming to identify proteins/metabolites that experienced significant changes before PLC, the potential impact of reverse causation and confounding variables still needs to be fully addressed. Methods: This study thoroughly investigated the causal relationship between 4719 blood proteins, 21 amino acids, and the risk of PLC using the Mendelian randomization (MR) method. In addition, through a comprehensive analysis of the TCGA-LIHC cohort and GEO databases, we evaluated the differentially expressed genes (DEGs) related to serine metabolism in diagnosing and predicting the prognosis of patients with PLC. Results: A total of 63 proteins have been identified as connected to the risk of PLC. Additionally, there has been confirmation of a positive cause–effect between PLC and the concentration of serine. The integration of findings from both MR analyses determined that the protein associated with PLC risk exhibited a significant correlation with serine metabolism. Upon careful analysis of the TCGA-LIHC cohort, it was found that eight DEGs are linked to serine metabolism. After thoroughly validating the GEO database, two DEGs, TDO2 and MICB, emerged as potential biomarkers for diagnosing PLC. Conclusions: Two proteins involved in serine metabolism, MICB and TDO2, are causally linked to the risk of PLC and could potentially be used as diagnostic indicators.

## 1. Introduction

Primary liver cancer, often known as PLC, is the sixth most prevalent form of cancer worldwide. It has the third-highest fatality rate among all malignant tumors globally [1]. PLC mainly consists of hepatocellular carcinoma (HCC), 75% to 85% of cases, and intrahepatic cholangiocarcinoma (ICC), accounting for 10% to 15% of cases. The primary causes of PLC include hepatitis virus infection, steatohepatitis, nonalcoholic fatty liver disease, and consumption of aflatoxin-contaminated food [2]. Alcohol-related PLC has the highest occurrence rate in Europe and the United States, comprising the most significant share [3]. China has the greatest prevalence of PLC associated with hepatitis B [4]. The pathogenesis of PLC is intricately linked to chronic liver inflammation, severe fibrosis, cirrhosis, and profound disruption of the liver microenvironment. On the one hand, long-term alcohol intake, obesity, and prolonged sitting considerably worsen chronic liver inflammation [5]. On the other hand, viral infection strongly stimulates immune cells, encourages the development of an inflammatory microenvironment, releases a substantial amount of cytokines, reactive oxygen species, and active nitrogen, and impacts cell metabolism and cycle, ultimately resulting in DNA damage and the creation of a tumor microenvironment [6]. The emergence of PLC is inconspicuous, its advancement is swift, and the management encounters the obstacles of medication resistance, metastasis, and recurrence. Despite the extensive use of immunotherapy, targeted therapy, radiation, chemotherapy, and other therapeutic modalities in the management of PLC, the survival rate of patients with advanced PLC is very low, leading to a dismal prognosis [7].

The fast advancement of mass spectrometry has led to the widespread use of proteomics and metabolomics in identifying illness causes and diagnostic indicators. The integration of proteomics and metabolomics enables the correlation of biomolecules across various functional pathways, thereby enhancing the comprehensiveness and reliability of in vivo reaction mechanism analysis [8,9]. Shang et al. [10] revealed that the levels of osteopontin in the blood of patients with HCC were dramatically increased using mass spectrometry. Osteopontin alone or combined with alpha-fetoprotein (AFP) demonstrated a high diagnostic accuracy for HCC. Du et al. [11] discovered the proteins expressed differently in tumor and nontumor tissue of patients with HCC. They then examined the proteins expressed differently in the serum of HCC patients, cirrhosis patients, and healthy volunteers. Aldo-keto reductase family 1 member B10 and cathepsin A were identified as promising biomarkers for the detection of HCC. Wei et al. [12] conducted a nontargeted metabolomics analysis on the serum of 26 patients with HCC and 26 healthy individuals. They identified 16 metabolites associated with nucleotide metabolism, exhibiting differential expression between the two groups. Additionally, they emphasized the potential diagnostic utility of genes involved in nucleotide metabolism for HCC. Nevertheless, most prior research was observational, so the findings were prone to confounding factors and restricted sample sizes. Additionally, most research has focused on proteins or metabolites whose expression changes in blood or tissues after PLC. The influence of reverse causation and confounding variables has yet to be entirely ruled out in studies attempting to identify proteins/metabolites that underwent substantial changes before PLC.

The randomized controlled trial (RCT) is the most dependable approach for investigating the causal relationship between risk factors and illnesses in epidemiological research. However, due to the lengthy study duration and significant use of people and material resources, achieving complete coverage of clinical issues may take time and effort. Katan first introduced the concept of Mendelian randomization (MR) as a statistical framework that utilizes genetic variation as an instrumental variable (IV) [13]. According to Mendel’s genetic laws, parents’ alleles are randomly allocated to children, which is analogous to the random grouping procedure in an RCT. Single-nucleotide polymorphisms (SNPs) are a form of DNA polymorphism. As the most frequent genetic variation, single-nucleotide polymorphisms (SNPs) are found throughout the human genome, accounting for more than 90% of all changes in human genomic DNA, with an average of one genotypic polymorphic SNP per thousand bases. MR uses SNPs to infer causal relationships between exposures and outcomes. Its benefit is that SNPs exist before acquired exposure, and the correlation with the outcome follows a causal, temporal relationship and is unaffected by acquired environmental, social, and other confounding factors [14]. Independent sample MR, two-sample MR, multivariate MR, and bidirectional MR extensively investigate the causal association between risk factors and diseases [15,16,17]. The MR method has proven the causal association between unhealthy lifestyle choices (such as alcohol use, smoking, and obesity) and HCC [18,19]. Drug MR methods have garnered considerable interest for their use in drug development and predicting medication effects. SNPs, specifically protein quantitative trait loci (pQTL) or expression quantitative trait loci (eQTL) that have substantial impacts on biomarkers near protein-coding genes of interest, are selected as IVs to investigate the causal effects of these protein targets on illnesses. This approach has promising potential for medication development [20,21].

Previously, a thorough review outlined the possible role of differently expressed proteins/metabolites in blood and urine in HCC diagnosis and prognosis [22]. However, as the authors point out in their paper, candidate markers face tests of reproducibility and heterogeneity [22]; we argue that most of the study they included was observational, and a range of confounding variables, including physiological and socioeconomic factors, influenced the findings. This study utilized extensive genome-wide association study (GWAS) data to examine the causal connection between blood proteome, amino acid, and PLC using the MR method. The objective is to minimize the impact of confounding variables and reverse causality, uncover early indicators associated with PLC risk, understand pathophysiology, and obtain novel viewpoints for identifying therapeutic targets for PLC.

## 2. Materials and Methods

### 2.1. Study Design of MR Analysis

The MR analysis is conducted strictly with the STROBE-MR checklist in Supplementary Table S1 [23]. The premise of MR analysis is that IVs need to satisfy three hypotheses: (1) IVs are strongly correlated with exposure factors; (2) there is no correlation between IVs and confounding factors; and (3) IVs can only affect outcomes by mediating exposure. In addition, MR analysis in this study mainly includes three steps: First, IVs strongly related to exposure were identified. Subsequently, various statistical methods, including inverse variance weighting (IVW) and MR-Egger regression, were used to evaluate the causal impact of exposure on the outcome. Finally, sensitivity analyses were performed to assess the validity of causal associations (primarily pleiotropy and the heterogeneity of IVs).

### 2.2. PLC, Proteome, and Metabolome Sample

We included 4719 blood protein data from the deCODE study [24]. PLC’s GWAS summary data from the FinnGen Consortium (https://www.finngen.fi/en, accessed on 24 January 2024) included 308 cases and 218,488 control samples. The 21 amino acids’ (alanine, arginine, asparagine, aspartate, cysteine, glutamine, glutamate, glycine, histidine, isoleucine, leucine, lysine, methionine, phenylalanine, proline, serine, threonine, tryptophan, tyrosine, valine, and citrulline) GWAS summary data were collected from the IEU database (https://gwas.mrcieu.ac.uk/, accessed on 24 January 2024).

### 2.3. IV Selection

In the introduction, we discussed the advantages of using SNPs as IVs for MR. On the one hand, SNPs exist before acquired exposure, ensuring that causal estimations are unaffected by reverse causality. Furthermore, SNPs are inherited separately from other features and are unaffected by other confounding variables [14]. As a result, we used the most recent and comprehensive population genetic variation data from publicly accessible sources for MR analysis. The fundamental assumptions of IVs and MR are mostly the same. The basic process of IV screening includes the following: (1) Deleting SNPs that do not fit the three vital assumptions. (2) Harmonizing SNPs whose exposure was consistent with the outcome. (3) Eliminating weak IVs with bias. To fulfill the three hypotheses of IVs, we specifically chose single SNPs that have a substantial association with plasma proteins (*p*-value < 5 × 10^−8^). Simultaneously, to mitigate the influence of linkage disequilibrium (LD) bias, SNPs that satisfied the following criteria were selected based on the European population dataset from the 1000 Genomes Project: r2 < 0.001 and genetic distance = 1000 kb. The screening criteria for IVs of the 21 amino acids and PLC were as follows: the significance criterion was set at a *p*-value of less than 5 × 10^−6^. The LD parameter was r2 < 0.001, and the genetic distance = 10,000 kb. To guarantee consistency, we harmonize IVs in both exposures and outcomes, ensuring they originate from DNA strands in the same direction. Furthermore, to determine the strength of the included SNPs as IVs, we computed the percentage of variation explained by each unique SNP and then calculated the F-statistic. SNPs with an F-statistic below 10 are eliminated to prevent potential bias in the causative evaluation. The information on all SNPs found after several screening processes is shown in Supplementary Table S2. These SNPs had F-statistics larger than 10, suggesting that the MR analysis findings were not influenced by weak IV bias.

### 2.4. MR Analysis

We primarily use the “TwoSampleMR” and “MR-PRESSO” packages in R (version 4.0.2) to perform analysis in our MR research. The odds ratio (OR) and 95% confidence interval (CI) were used to quantify the association, and a *p*-value less than 0.05 was considered statistically significant. IVW is a fundamental analytical approach that estimates the causal impact. The IVW assumes that all SNPs fulfill the three basic requirements of MR, analyzes the Wald ratio between exposure and outcome for each SNP, and then combines the data to obtain the causal effect value [25]. The IVW method provides excellent estimate accuracy and testing efficiency when IVs lack pleiotropy. However, IVW methods are subject to weak tool bias and pleiotropy. Bowden et al. developed MR-Egger to address this issue, which measures average pleiotropy between IVs using an intercept term [26]. The MR-Egger method can still provide an unbiased causal estimate even if certain IVs exhibit pleiotropy. The MR-Egger approach is often employed to assess the genetic pleiotropy of IVs. The weighted median method gives an accurate causal estimate based on the assumption that at least 50% of IVs are valid. The simple model, weighted median, weighted model, and MR-Egger methods are used to address the limitations of the IVW method. The causal relationship was considered significant if the *p*-value of the causal association, determined using the IVW approach, reached statistical significance, and the estimated direction of causality was consistent with that of the other four methods.

Pleiotropy is when a single genetic locus may influence many observable characteristics, known as phenotypes. This phenomenon can substantially impact the accuracy and dependability of MR analysis findings. We used the MR-Egger intercept test to evaluate the presence of a possible pleiotropy of SNPs. Furthermore, we used Cochran’s Q test to assess the heterogeneity across SNPs. If Cochran’s Q test was statistically significant (*p*-value < 0.05 and I^2^ > 25%), it indicated significant heterogeneity among SNPs [27]. The MR–Pleiotropic Residuals and Outliers (MR-PRESSO) is the primary method for testing horizontal pleiotropy, but its application conditions are harsh, and at least 50% of IVs must be effective. The MR-PRESSO method determines the presence of statistically significant outliers in the MR analysis. The leave-one-out (LOO) approach has also been used to assess the magnitude of causal estimates for a single IV. The LOO approach removes each SNP in turn then recalculates the MR result using the remaining SNPs. If there is no substantial difference between the eliminated and full MR results, the MR results are reliable.

### 2.5. Identification of Differentially Expressed Genes (DEGs)

The Cancer Genome Atlas Program (TCGA) database (https://www.cancer.gov/ccg/research/genome-sequencing/tcga, accessed on 24 January 2024) was queried for RNA-seq data related to 374 HCC tumor tissues and 50 adjacent tissues, in addition to general and survival information regarding patients. GSE62232 (platform GPL570, comprising 81 HCC samples and 10 normal samples) and GSE101685 (platform GPL570, containing 24 HCC samples and 8 normal samples) data were acquired from the GEO database (https://www.ncbi.nlm.nih.gov/geo/, accessed on 24 January 2024). The screening criteria for DEGs is that the absolute value of Log2Foldchange is greater than 1 and the adjusted *p*-value is less than 0.05. Genes related to serine metabolism were obtained from the Genecards database (https://www.genecards.org/, accessed on 24 January 2024).

### 2.6. Enrichment Analysis

The positive results obtained from MR analysis were incorporated into the DAVID database (https://david.ncifcrf.gov/home.jsp, accessed on 24 January 2024) to conduct Gene Ontology (GO) and Kyoto Encyclopaedia of Genes and Genomes (KEGG) enrichment analyses. GO enrichment analysis includes biological processes, cellular components, and molecular functions. Term significance is determined by a *p*-value less than 0.05, and bubble plots are generated accordingly.

### 2.7. Analysis of Immune Infiltration

We used the 24 immune cell markers contributed by Bindea [28] to calculate the immune cell infiltration in the TCGA-LIHC cohort sequencing data using the ssGSEA method. The resulting correlation between the immune infiltration matrix and individual gene data was then displayed using the “ggplot2 package”.

### 2.8. Diagnosis and Prognosis Analysis

The Kaplan–Meier method was employed to analyze survival data, while receiver operating characteristic (ROC) curves were utilized to assess the predictive value of various genetic diagnoses in patients. A difference is considered statistically significant when the *p*-value is less than 0.05.

## 3. Results

### 3.1. Causal Effects of Proteome on PLC

**Figure 1:**
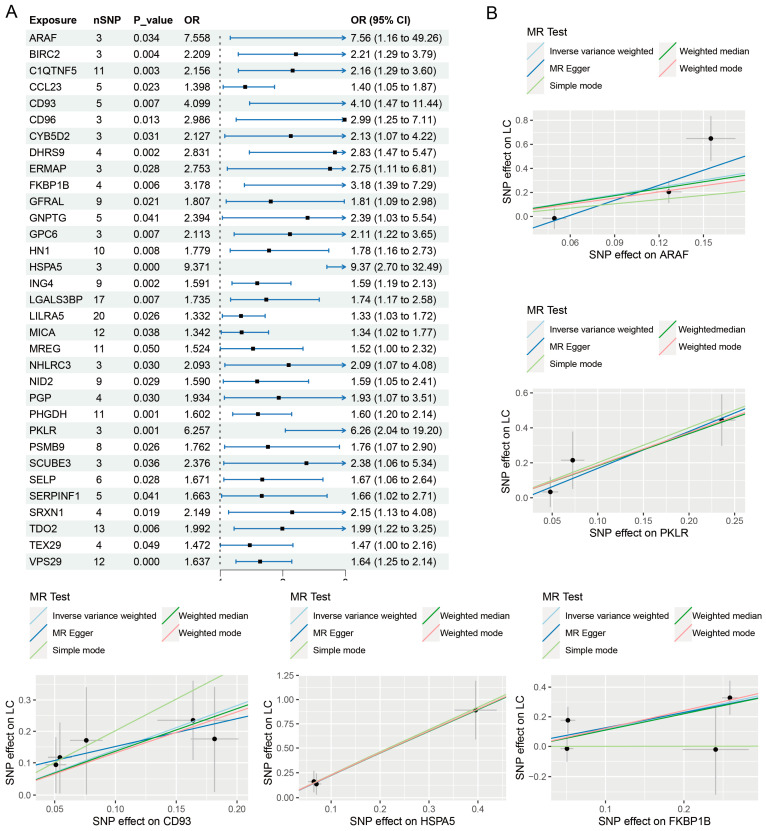
Causal effects of plasma protein on PLC risk (positive correlation). Forest plot (**A**) and partial scatter plots (**B**) of plasma protein and PLC risk.

**Figure 2:**
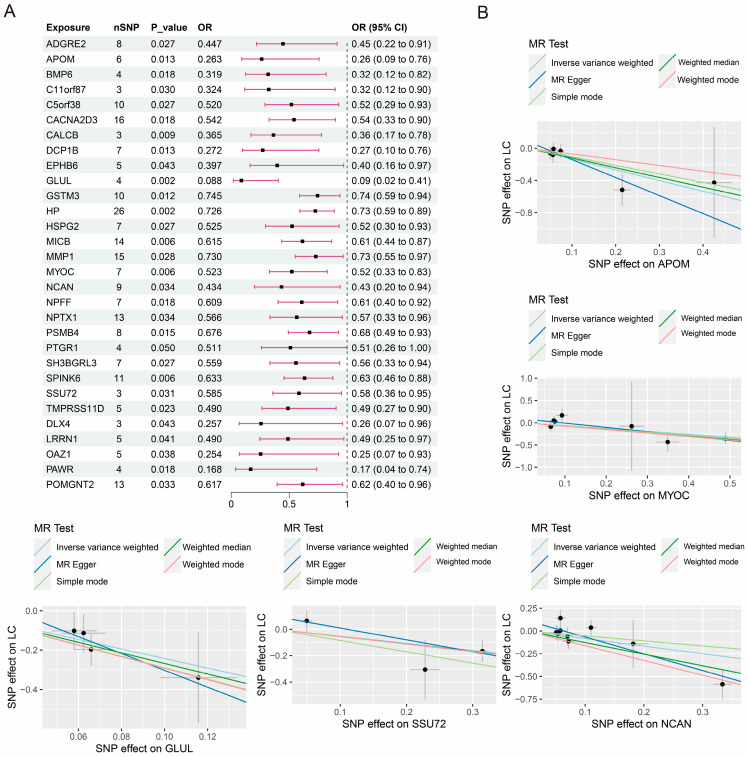
Causal effects of plasma protein on PLC risk (negative correlation). Forest plot (**A**) and partial scatter plots (**B**) of plasma protein and PLC risk.

Using the IVW approach, it was determined that 33 proteins exhibited a positive correlation with the risk of PLC, as shown in Figure 1. Additionally, 30 proteins had a negative correlation with the risk of PLC, as shown in Figure 2. The findings obtained from the simple model, weighted median, weighted model, and MR-Egger methods all demonstrate consistent causal estimating trends. Cochran’s Q test did not reveal any heterogeneity, as shown in Table 1. The MR-Egger intercept test could not detect any evidence of horizontal pleiotropy (*p*-value > 0.05). The funnel plots display the findings of the heterogeneity test conducted during the MR analysis (Supplementary Figure S1). Furthermore, the MR-PRESSO global test did not detect any outliers. The LOO analysis results demonstrate that a single SNP does not influence the MR analysis findings (Supplementary Figure S2). Ultimately, a sequence of sensitivity analysis indicates that the MR results obtained from this research are reliable.

### 3.2. Causal Effects of Amino Acid and PLC

**Figure 3:**
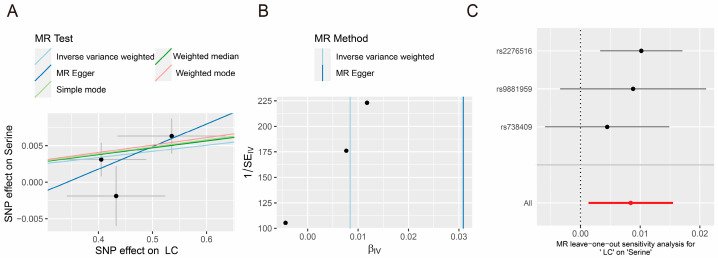
Causal effects of amino acid and PLC. The scatter plot (**A**), funnel plot (**B**), and forest plot analyzed by LOO analysis (**C**) of PLC on serine concentration.

The IVW approach did not demonstrate any causal relationship between amino acids and the risk of PLC. The reverse MR analysis revealed that PLC had a statistically significant positive causal impact on serine concentration (OR = 1.008, 95% CI = 1.001–1.016, *p*-value = 0.02). The findings obtained from the simple model, weighted median, weighted model, and MR-Egger methods all demonstrate consistent causal estimating trends (Figure 3A). Cochran’s Q test did not reveal any heterogeneity (*p*-value = 0.276). The MR-Egger intercept test could not detect any evidence of horizontal pleiotropy (*p*-value > 0.05). The funnel plots display the findings of the heterogeneity test conducted during the MR analysis (Figure 3B). Furthermore, the MR-PRESSO global test did not detect any outliers (*p*-value = 0.297). The LOO analysis results demonstrate that a single SNP does not influence the MR analysis findings (Figure 3C). Ultimately, a sequence of sensitivity analysis indicates that the MR results obtained from this research are reliable.

### 3.3. Identification of TCGA-LIHC Cohort-Related DEGs and Enrichment Analysis

**Figure 4:**
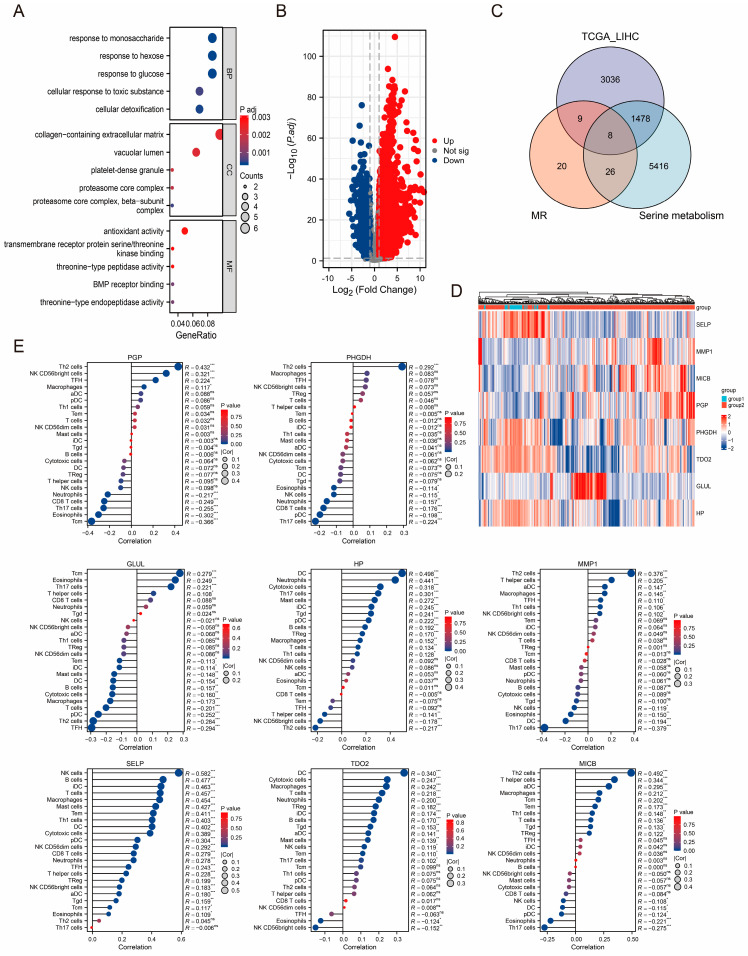
Identification of TCGA-LIHC cohort-related DEGs and enrichment analysis. (**A**). GO enrichment analysis of positive results of MR analysis. (**B**). Volcano plot of DEGs in the TCGA-LIHC cohort. (**C**). Venn diagram of serine metabolism-related genes, DEGs in TCGA-LIHC cohort, and positive results of MR analysis. (**D**). Heat map of the expression of 8 key DEGs in the TCGA-LIHC cohort. (**E**). Lollipop chart for correlation analysis of 8 key DEGs and immune cell infiltration. *, ** and *** are expressed as *p*-Values less than 0.05, 0.01 and 0.001, respectively. ns indicates no statistical significance.

Initially, we conducted GO and KEGG enrichment analysis on 63 positive proteins. The results showed that risk proteins are primarily located in the collagen-containing extracellular matrix, vacuolar lumen, and platelet-dense granule, involving molecular functions, such as antioxidant activity, transmembrane receptor protein serine/threonine kinase binding, and threonine-type peptidase activity, and participating in biological processes, such as response to monosaccharide, response to hexose, and response to glucose (see Figure 4A). It is important to note that the KEGG enrichment analysis yielded just one significant term, “Biosynthesis of amino acids”, with a *p*-value of 0.029. By integrating the findings from both MR analyses, we established that the protein linked to PLC risk strongly correlated with serine metabolism. Following that, we detected 3299 upregulated and 1232 downregulated genes using the TCGA-LIHC cohort (Figure 4B). A total of 6928 genes associated with serine metabolism were discovered from the Genecards database. Figure 4C displays eight DEGs, including glutamine synthetase (GLUL), interstitial collagenase (MMP1), haptoglobin (HP), tryptophan 2,3-dioxygenase (TDO2), MHC class I polypeptide-related sequence B (MICB), glycerol-3-phosphate phosphatase (PGP), P-selectin (SELP), and D-3-phosphoglycerate dehydrogenase (PHGDH), associated with serine metabolism. The expression of these eight DEGs in HCC tissues exhibited considerable heterogeneity compared to adjacent tissues (Figure 4D). In addition to metabolic reprogramming, immune cell infiltration is a significant characteristic of PLC development. We used the ssGSEA technique to assess the infiltration of immune cells in HCC samples. Subsequently, the findings indicated a substantial correlation between those above eight DEGs and the infiltration of immune cells, as shown in Figure 4E.

### 3.4. Diagnostic and Prognostic Value of DEGs in TCGA-LIHC Cohort

**Figure 5:**
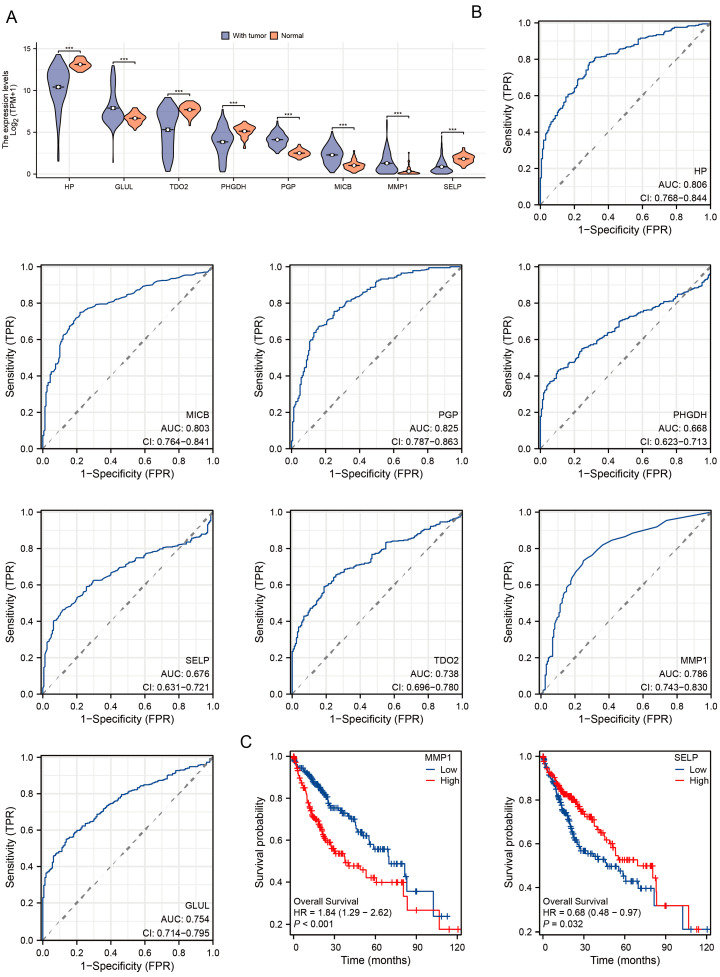
Diagnostic and prognostic value of 8 key DEGs in TCGA-LIHC cohort. (**A**). Violin plot of 8 key DEGs expression in TCGA-LIHC cohort. (**B**). ROC curves of 8 key DEGs. (**C**). Kaplan–Meier plots of 2 key DEGs. *** indicates a *p*-Value less than 0.001.

Figure 5A illustrates the disparity in the expression of eight DEGs between tumor and paracancerous tissues. The markers GLUL, MMP1, HP, TDO2, MICB, and PGP have diagnostic significance for HCC, as shown in Figure 5B. Two DEGs, namely MMP1 and SELP, provide significant potential for assessing the prognosis of HCC, as seen in Figure 5C.

**Figure 6:**
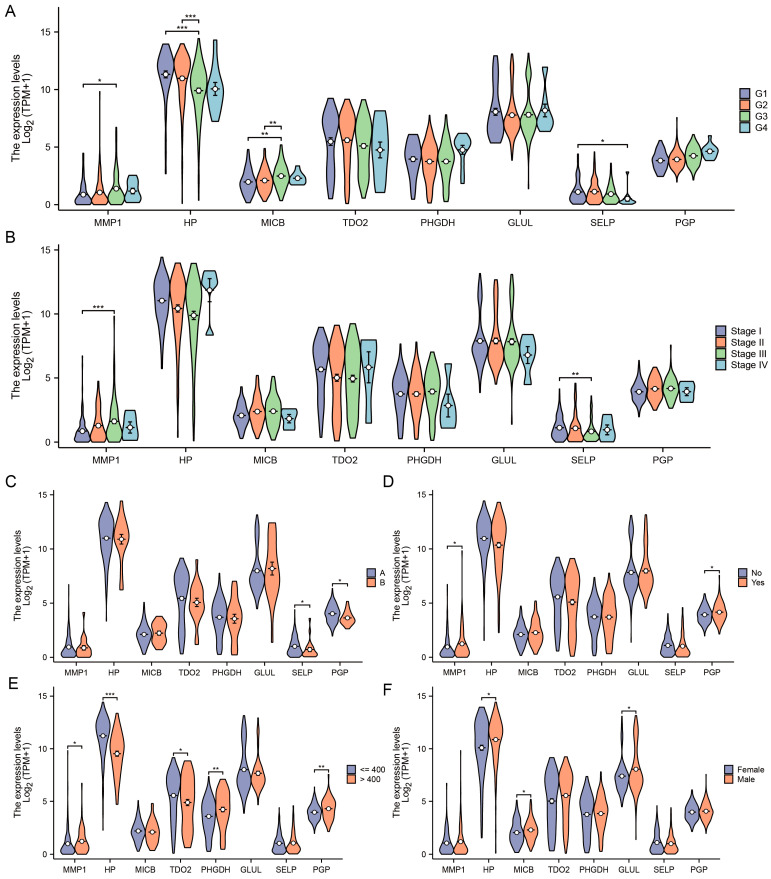
Correlation analysis of 8 key genes and clinical features. Comparison of expression levels of 8 key DEGs with histologic grade (**A**), TMN stage (**B**), Child–Pugh grade (**C**), vascular invasion (**D**), AFP (**E**), and gender (**F**). *, ** and *** are expressed as *p*-Values less than 0.05, 0.01 and 0.001, respectively.

When considering additional clinical indicators, we observed a substantial correlation between MMP1, HP, MICB, and SELP expressions and histologic grade (Figure 6A). The expression of SELP and MMP1 showed a significant correlation with the TNM stage, as seen in Figure 6B. The levels of SELP and PGP showed a strong correlation with the Child–Pugh grade (Figure 6C). The levels of PGP and MMP1 were strongly associated with vascular invasion, as seen in Figure 6D. The MMP1, HP, TDO2, PHGDH, and PGP levels showed a strong correlation with AFP, as seen in Figure 6E. The GLUL, MICB, and HP expressions showed a strong correlation with gender, as shown in Figure 6F.

**Figure 7:**
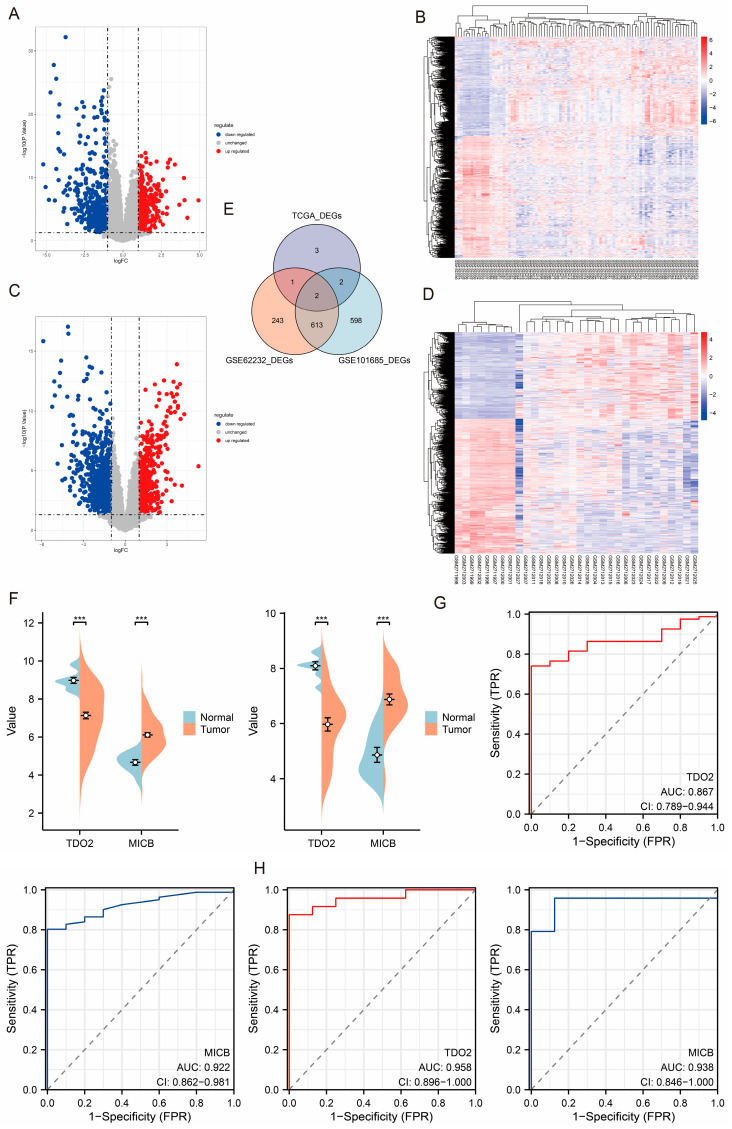
Volcano plot (**A**) and heat map (**B**) of GSE62232 cohort-related DEGs. Volcano plot (**C**) and heat map (**D**) of GSE101685 cohort-related DEGs. (**E**). Venn diagram of DEGs for 8 key DEGs and GSE62232 and GSE101685 cohort. (**F**). Bean plot of TDO2 and MICB expression in GSE62232 and GSE101685 cohort. ROC curves of TDO2 and MICB in GSE62232 (**G**) and GSE101685 (**H**) cohort. *** indicates a *p*-Value less than 0.001.

We conducted further tests utilizing the GSE62232 and GSE101685 datasets as external validation sets. The GSE62232 dataset revealed the presence of 386 upregulated and 473 downregulated genes (Figure 7A). Additionally, the gene expression patterns of tumor and normal tissues exhibited substantial heterogeneity (Figure 7B). The GSE101685 dataset identified 475 upregulated and 740 downregulated genes (Figure 7C). Additionally, the gene expression patterns of tumor and normal tissues exhibited notable heterogeneity (Figure 7D). Two genes, TOD2 and MICB, were found to be shared, as shown in Figure 7E,F. The findings of the ROC curve indicate that TDO2 and MICB may have diagnostic significance in patients with HCC (Figure 7G,H).

## 4. Discussion

The first manifestations of PLC are inconspicuous, leading to a delayed diagnosis in most patients who have already progressed to an advanced stage. Surgical interventions, chemotherapy, and radiation provide unsatisfactory results, resulting in a bleak prognosis [7]. Clinicians have used techniques such as proteomics and metabolomics to identify proteins or metabolites that may have diagnostic significance [8,9]. Lu et al. used blood samples from 46 hepatitis B virus-associated HCC patients with cirrhosis and 24 healthy volunteers for metabolomic study. They discovered that palmitoylcarnitine and arginine may be valuable markers for identifying hepatitis B virus-associated HCC patients [29]. Di et al. found the effectiveness of various race-specific metabolites in identifying HCC patients [30]. Zou et al. investigated the differences in metabolite expression patterns between male and female HCC patients [31]. The findings revealed that dysregulation of metabolite expression was more severe in female HCC patients than in males. Abnormal amino acid metabolism has also been linked to the development of HCC [32]. Cao et al. employed next-generation sequencing technology and machine learning approaches to discover that mutations at specific amino acid sites might cause HCC [32]. Previous research examined the expression of 2761 metabolism-related genes in HCC tissues and assessed the predictive value of particular genes in HCC prognosis [33]. Proteomic approaches, such as quantitative proteomic methods based on iTRAQ, have been utilized to identify diagnostic markers for HCC [34]. Using mass spectrometry, Sun et al. previously discovered 116 protein markers that differentiate HCC from normal hepatocytes. They then gathered serum samples and clinical data from HCC patients for the study. They found that tissue transglutaminase 2 might be a promising histological/serum protein biomarker for HCC diagnosis, particularly for identifying HCC patients with normal serum AFP expression [35]. Interestingly, Ozawa et al. discovered substantial changes in dipeptide patterns between HCC and nontumor tissues [36]. Uzzaman et al. extracted extracellular vesicles from the serum of HCC patients, cirrhotic patients, and healthy volunteers. They discovered that extracellular vesicle-specific proteins such as thrombospondin-1, fibulin-1, and fibrinogen gamma chain could distinguish between healthy volunteers and patients with cirrhosis and HCC [37].

Nevertheless, no studies have successfully identified the specific proteins or metabolites that play a role in the initiation of PLC while ruling out the influence of confounding factors and reverse causation. Using MR methods in this work, a causal relationship between 63 proteins and PLC risk was found. The KEGG enrichment analysis revealed that 63 risk proteins had significant enrichment only in the amino acid biosynthesis pathway. Consequently, we employed MR analysis to investigate the bidirectional causal relationship between amino acids and PLC. The results showed no causal relationship between amino acids and PLC risk. Reverse causal analysis indicated that PLC only affects serine concentration, suggesting an abnormal change in serine metabolism following PLC occurrence. Therefore, we hypothesized that serine metabolism plays a crucial role in influencing PLC risk among the 63 proteins, and the changes in serine concentration after PLC occurrence may be closely related to tumor development. A thorough investigation of the TCGA and GEO databases showed that serine metabolism-related DEGs (such as TDO2 and MICB) had significant diagnostic value in PLC.

Metabolic reprogramming is a much-discussed subject in cancer research [38]. Previously, it has been shown that the Warburg effect, which refers to glucose breaking down into pyruvate and then converting into lactic acid in tumor cells without undergoing aerobic oxidation in the mitochondria, is a characteristic of most tumor cells [39]. The MR study revealed a total of 63 proteins that were directly linked to the risk of PLC. The KEGG enrichment analysis of 63 proteins determined that only the term “biosynthesis of amino acids” showed a statistically significant association. Consequently, we conducted a more thorough assessment of the bidirectional MR analysis between 21 amino acids and the risk of PLC. We have discovered a clear and direct link between PLC and serine concentrations, with a positive causal correlation (OR = 1.008, 95% CI = 1.001–1.016, *p*-value = 0.02). Previous research also corroborates our perspective that serine metabolism experiences substantial activation during the progression of PLC [40]. Serine serves as a central point for several crucial metabolic processes and controls immunological function, inflammatory response, and cell development and proliferation. Tumor cells may experience an increase in their antioxidant and methylation capability due to the buildup of serine [41]. Labuschagne et al. [42] reported that tumor cells need a significant quantity of serine to sustain their proliferation, and the consumption of serine may impede the growth of tumor cells. In a recent study, Li et al. [43] found that remodeling serine metabolism improves the sensibility of HCC cells to sorafenib, a primary medication for HCC patients who are not eligible for surgery or have distant metastasis. PHGDH is a crucial serine biosynthesis enzyme. Research has shown that PHGDH is excessively produced in HCC cells, and suppressing PHGDH expression may substantially impact cancer cell proliferation, migration, and invasion [44]. Increasing the ubiquitination degradation of PHGDH may effectively suppress the growth of HCC cells and the characteristics of cancer stem cells [45]. Shu et al. [46] discovered that PHGDH may facilitate the proliferation, migration, and invasion of HCC cells by enhancing the synthesis of proteins encoded by mitochondrial DNA and mitochondrial respiration. Our MR analysis indicated that PHGDH is strongly associated with an elevated risk of PLC (OR = 1.602, 95% CI = 1.20–2.14, *p*-value = 0.029). Ultimately, the onset and progression of PLC are significantly influenced by the crucial role played by PHGDH in the aberrant serine metabolism.

Following MR research to establish the relationship between serine metabolism and PLC risk, we thoroughly analyzed RNA sequencing data from the TCGA-LIHC cohort. Our research revealed eight DEGs connected to serine that were causally linked to the risk of PLC. GLUL, MMP1, HP, TDO2, MICB, and PGP have significant diagnostic potential for PLC since their AUC values are above 0.7. The eight DEGs were also strongly correlated with dendritic cells (DC), macrophages, and NK cell infiltration during PLC. Furthermore, the eight DEGs are strongly associated with several clinical parameters of patients with PLC, such as histologic grade, TMN stage, Child–Pugh grade, vascular invasion, gender, and AFP concentration. It is worth mentioning that MMP1 and SELP have significant implications for the prognostic evaluation of PLC. Subsequently, we used the GSE62232 and GSE101685 datasets from the GEO database to validate the expression patterns of these eight genes in tumor tissues. This analysis identified two crucial DEGs (TDO2 and MICB).

TDO2 is a crucial rate-limiting enzyme in tryptophan (TRP) metabolism. Elevated TDO2 activity increases the concentration of kynurenine (Kyn) by reducing the amount of TRP in the nearby microenvironment. Kyn may undergo catalysis by kynurenine aminotransferase to yield kynurenic acid. The metabolites above can impede the immune response by stimulating the aromatic hydrocarbon receptor, hence facilitating the advancement of tumors [47]. Furthermore, T cells have a heightened sensitivity to minimal amounts of TRP, and such low levels of TRP may trigger a response of amino acid deprivation, leading to the demise of T cells and the promotion of immune evasion [48]. The results of our investigation indicate a direct link between TDO2 and the risk of HCC (OR = 1.99, 95% CI = 1.22–3.25, *p*-value = 0.006). The result aligns with the conclusions found in several prior investigations. The expression of TDO2 is positively associated with worse clinicopathological characteristics and prognosis in individuals with HCC [49]. Both in vivo and in vitro experiments have verified that increased TDO2 expression can significantly facilitate the onset and progression of HCC. This effect may be attributed to the activation of aromatic hydrocarbon receptors and the promotion of the IL-6-STAT3 signaling pathway [50]. Our thorough examination of two queues in the GEO database further confirms the diagnostic capabilities of TDO2 (GSE62232: AUC = 0.867; GSE101685: AUC = 0.958). Nevertheless, there is conflicting evidence about the pattern of TDO2 expression in individuals with HCC. From a physiological standpoint, TDO2 is primarily located in hepatocytes and neurons. Through differential expression analysis of the TCGA-LIHC and GEO cohort, we observed a substantial decrease in TDO2 expression in the tissues of patients with HCC. Prior single-center clinical investigations have shown that TDO2 expression increases in individuals with HCC [49]. This discrepancy may be due to variations in ancestral populations or clinical stage standards.

NKG2D is crucial in activating natural killer (NK) cells. MICA/B serve as the primary ligands for NKG2D, and together, they create a functional ligand–receptor complex involved in immune surveillance [51]. During the early phase of tumor cell formation, activating the MICB/NKG2D signaling pathway leads to the release of several cytokines. This, in turn, stimulates immune cells like dendritic cells, facilitating their involvement in the adaptive immune response. Upregulating MICB expression may significantly augment the immunogenicity of NK cells, leading to enhanced immune-mediated cytotoxicity [51]. However, during the latter stages of tumor growth, there is a notable drop in the expression of MICB/NKG2D. This decrease hampers the ability of NK cells to effectively destroy tumor cells, potentially leading to immune evasion [51]. Our MR study revealed that MICB can decrease the likelihood of HCC with an OR of 0.615, a 95% CI ranging from 0.44 to 0.87, and a *p*-value of 0.006. A thorough examination of the TCGA-LIHC and GEO cohort revealed that MICB expression was notably increased in tumor tissues compared to normal tissues. The finding also suggests that MICB can be used as a diagnostic tool. Our thorough examination of two queues in the GEO database further confirms the diagnostic capabilities of MICB (GSE62232: AUC 0.922; GSE101685: AUC = 0.938). Fang et al. [52] discovered that the expression of MICA/B was strongly and inversely associated with the extent of lymph node metastasis. They have verified that the unfolded protein response may serve as a method for downregulating the expression of MICA/B. Furthermore, Li et al. [53] validated that the elevated expression of NLR family pyrin domain containing 3 (NLRP3) in HCC might hinder the interaction between NKG2D and MICA. Following the NLRP3 deletion, there was a notable rise in MICB expression, resulting in an augmentation of the immunotoxicity of NK cells. Our work concludes that targeting MICB might effectively lower the likelihood of developing HCC and potentially serve as a diagnostic marker for HCC.

MR approach evaluation of causal relationships between exposure and outcomes is a potent technique. In contrast to observational studies, MR analysis reduces the likelihood of confounding and reverse causation-induced bias. Our MR study rigorously examined the causal relationship between 4719 blood proteins, 21 amino acids, and PLC risk by the general STROBE-MR guidelines. Simply put, we incorporated the most exhaustive GWAS data and screened for dependable IVs via procedures including significance threshold setting, elimination of linkage disequilibrium, and confounding factor elimination. The causal relationships between exposures and factors were evaluated using five distinct methodologies. Additionally, none of the results detected heterogeneity and horizontal pleiotropy of IVs. Furthermore, by thoroughly examining the TCGA and GEO databases, we assessed the diagnostic and prognostic significance of genes associated with serine metabolism in patients with HCC. The outcomes of our study provide viable approaches for the clinical diagnosis, prognosis evaluation, and target identification in patients with HCC. Nevertheless, there are several constraints inherent in this research. Initially, all of the GWAS data included in our study originated only from European populations. Consequently, the reliability and accuracy of our findings about other populations require further confirmation. Furthermore, the number of patients included in PLC’s GWAS is limited, and it is necessary to augment the sample size in future studies to provide additional substantiation for the findings.

## 5. Conclusions

This research thoroughly evaluates the possible causative connection between blood proteins, amino acids, and PLC. We have found 63 proteins linked to an increased risk of PLC. KEGG enrichment analysis of the 63 proteins revealed significant enrichment only in the amino acid biosynthesis pathway, enhancing the understanding of the connection between blood proteins and amino acids in the development of liver cancer. The bidirectional MR analysis between amino acids showed no causal relationship between amino acids and PLC risk, while PLC only affects serine concentration. A thorough examination of the TCGA-LIHC database revealed eight DEGs associated with serine metabolism. Upon further validation of the GEO database, two DEGs, TDO2 and MICB, were identified as possible biomarkers for diagnosing PLC. TDO2 and MICB participate in the development of PLC by regulating serine metabolism and may be targets for drug development.

## Figures and Tables

**Table 1:** Assessing the heterogeneity and pleiotropy of causal effects of plasma protein on PLC risk.

Exposure	Cochran’s Q Test		Horizontal Pleiotropy		MR-PRESSO
	I^2^	*p*-Value	Egger Intercept	*p*-Value	*p*-Value
ADGRE2	0	0.973	−0.024	0.742	0.976
APOM	0	0.766	0.083	0.377	0.682
ARAF	62%	0.072	−0.240	0.489	NA
BIRC2	4%	0.353	−0.135	0.413	0.785
BMP6	0	0.954	0.053	0.759	0.601
C11orf87	0	0.924	0.007	0.961	NA
C1QTNF5	16%	0.294	−0.061	0.491	0.304
C5orf38	27%	0.199	0.049	0.383	0.202
CACNA2D3	2%	0.425	0.019	0.702	0.524
CALCB	0	0.733	0.053	0.640	0.904
CCL23	0	0.957	−0.148	0.794	0.963
CD93	0	0.967	0.062	0.596	0.994
CD96	0	0.993	−0.263	0.925	0.810
CYB5D2	0	0.775	0.057	0.606	0.315
DCP1B	0	0.467	0.252	0.572	0.413
DHRS9	0	0.400	−0.090	0.441	0.447
EPHB6	34%	0.195	0.047	0.834	0.388
ERMAP	0	0.786	−0.077	0.615	0.697
FKBP1B	13%	0.329	0.023	0.851	0.539
GFRAL	0	0.495	−0.069	0.418	0.606
GLUL	0	0.904	0.125	0.704	0.891
GNPTG	0	0.616	−0.074	0.492	0.599
GPC6	0	0.572	−0.099	0.501	0.056
GSTM3	0	0.568	−0.109	0.168	0.588
HN1	0	0.919	0.041	0.472	0.856
HP	0	0.830	−0.013	0.771	0.817
HSPA5	0	0.973	−0.003	0.983	NA
HSPG2	0	0.527	−0.030	0.664	0.59
ING4	0	0.740	−0.022	0.754	0.688
LGALS3BP	21%	0.213	−0.034	0.467	0.247
LILRA5	0	0.644	0.010	0.766	0.762
MICA	0	0.795	0.105	0.332	0.828
MICB	0	0.770	0.031	0.592	0.827
MMP1	0	0.612	−0.010	0.817	0.393
MREG	8%	0.370	−0.043	0.621	0.573
MYOC	0	0.489	0.088	0.218	0.439
NCAN	29%	0.184	0.117	0.073	0.159
NHLRC3	0	0.749	−0.087	0.594	0.683
NID2	0	0.497	0.022	0.769	0.542
NPFF	0	0.877	−0.017	0.797	0.885
NPTX1	0	0.983	−0.005	0.918	0.985
PGP	0	0.886	0.048	0.893	0.764
PHGDH	0	0.718	−0.047	0.591	0.712
PKLR	0	0.723	−0.043	0.760	NA
PSMB4	0	0.874	−0.061	0.450	0.926
PSMB9	0	0.492	−0.027	0.686	0.633
PTGR1	0	0.942	−0.035	0.904	0.953
SCUBE3	0	0.999	0.005	0.971	NA
SELP	0	0.919	0.071	0.469	0.467
SERPINF1	0	0.450	0.014	0.922	0.515
SH3BGRL3	3%	0.400	−0.033	0.657	0.615
SPINK6	5%	0.395	−0.205	0.203	0.382
SRXN1	0	0.997	0.019	0.934	0.925
SSU72	0	0.396	0.098	0.497	0.824
TDO2	0	0.809	−0.058	0.422	0.721
TEX29	0	0.687	−0.443	0.447	0.534
TMPRSS11D	1%	0.400	0.094	0.385	0.551
VPS29	0	0.548	−0.025	0.639	0.483

## Data Availability

GWAS data were used for analysis in the present study, as well as data from the FinnGen database (https://www.finngen.fi/en, accessed on 1 January 2023) and the IEU (https://gwas.mrcieu.ac.uk/, accessed on 1 January 2023) publicly accessible database.

## Supplementary Materials

The following supporting information can be downloaded at: https://www.mdpi.com/article/10.3390/jpm14030262/s1, Supplementary tables: Table S1. STROBE-MR checklist; Table S2. SNPs information after harmonization. Supplementary figures: Partial funnel plots (Figure S1), and LOO analysis (Figure S2) for the causal effect of protein on the risk of PLC.

## Abbreviations

PLC: primary liver cancer; MR: Mendelian randomization; DEGs: differentially expressed genes; HCC: hepatocellular carcinoma; ICC: intrahepatic cholangiocarcinoma; AFP: alpha-fetoprotein; RCT: randomized controlled trial; IV: instrumental variable; SNPs: single-nucleotide polymorphisms; QTL: quantitative trait loci; GWAS: genome-wide association study; IVW: inverse variance weighting; LD: linkage disequilibrium; OR: odds ratio; CI: confidence interval; MR-PRESSO: MR-pleiotropic residuals and outliers; LOO: leave-one-out; KEGG: Kyoto Encyclopedia of Genes and Genomes; GO: Gene Ontology; ROC: receiver operating characteristic; GLUL: glutamine synthetase; MMP1: interstitial collagenase; HP: haptoglobin; TDO2: tryptophan 2,3-dioxygenase; MICB: MHC class I polypeptide-related sequence B; PGP: glycerol-3-phosphate phosphatase; SELP: P-selectin; PHGDH: D-3-phosphoglycerate dehydrogenase; TRP: tryptophan; Kyn: kynurenine; NK: natural killer; NLRP3: NLR family pyrin domain containing 3.
