# Supplementary figures and images for: Identifying Proteins and Amino Acids Associated with Liver Cancer Risk: A Study Utilizing Mendelian Randomization and Bulk RNA Sequencing Analysis
Source: J Pers Med. 2024 Feb 28;14(3):262. doi: 10.3390/jpm14030262 (PMC10970996; doi:10.3390/jpm14030262)

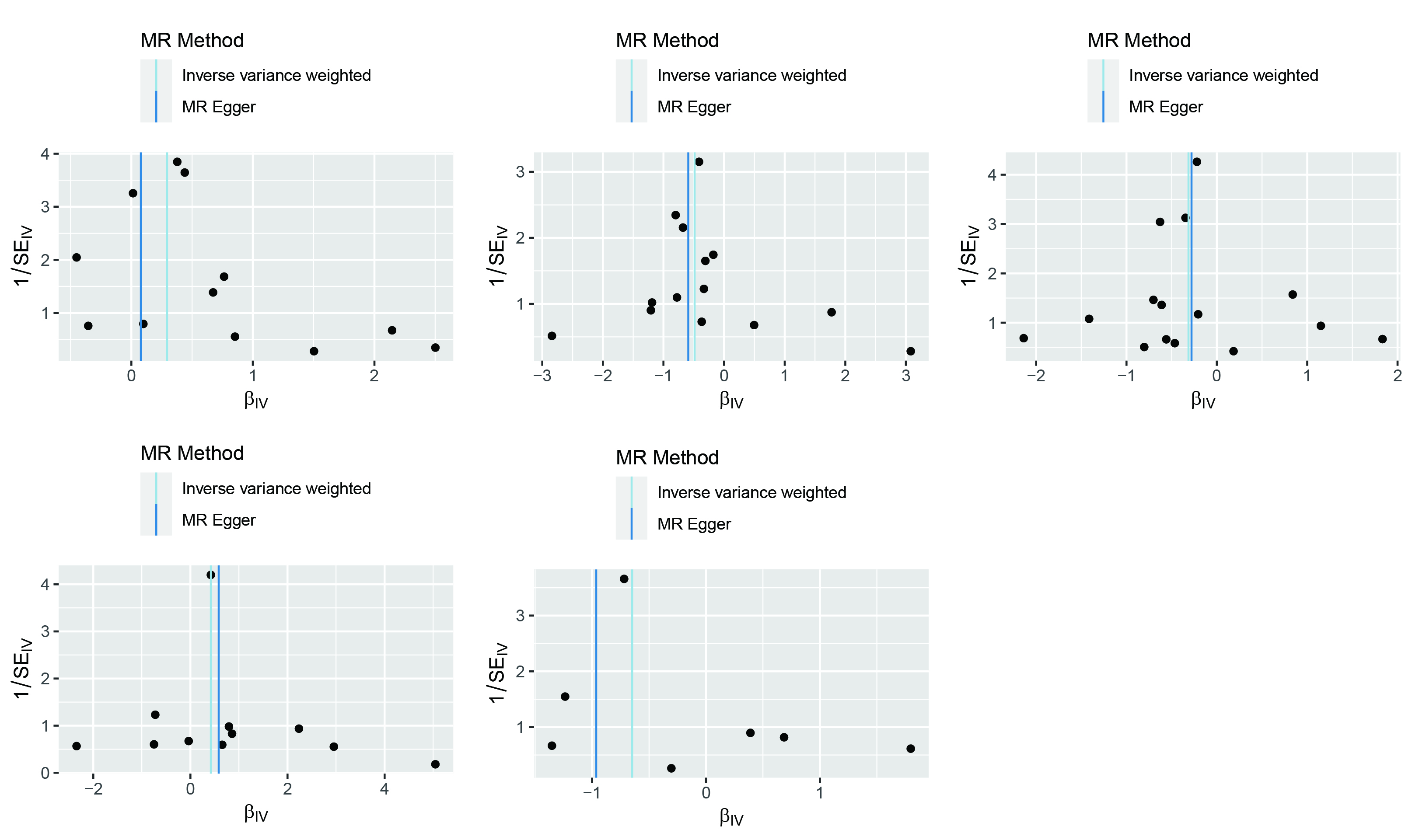

Supplement: Supplementary file 1 [file jpm-14-00262-s001.zip › Supplementary Figure S1.jpg]

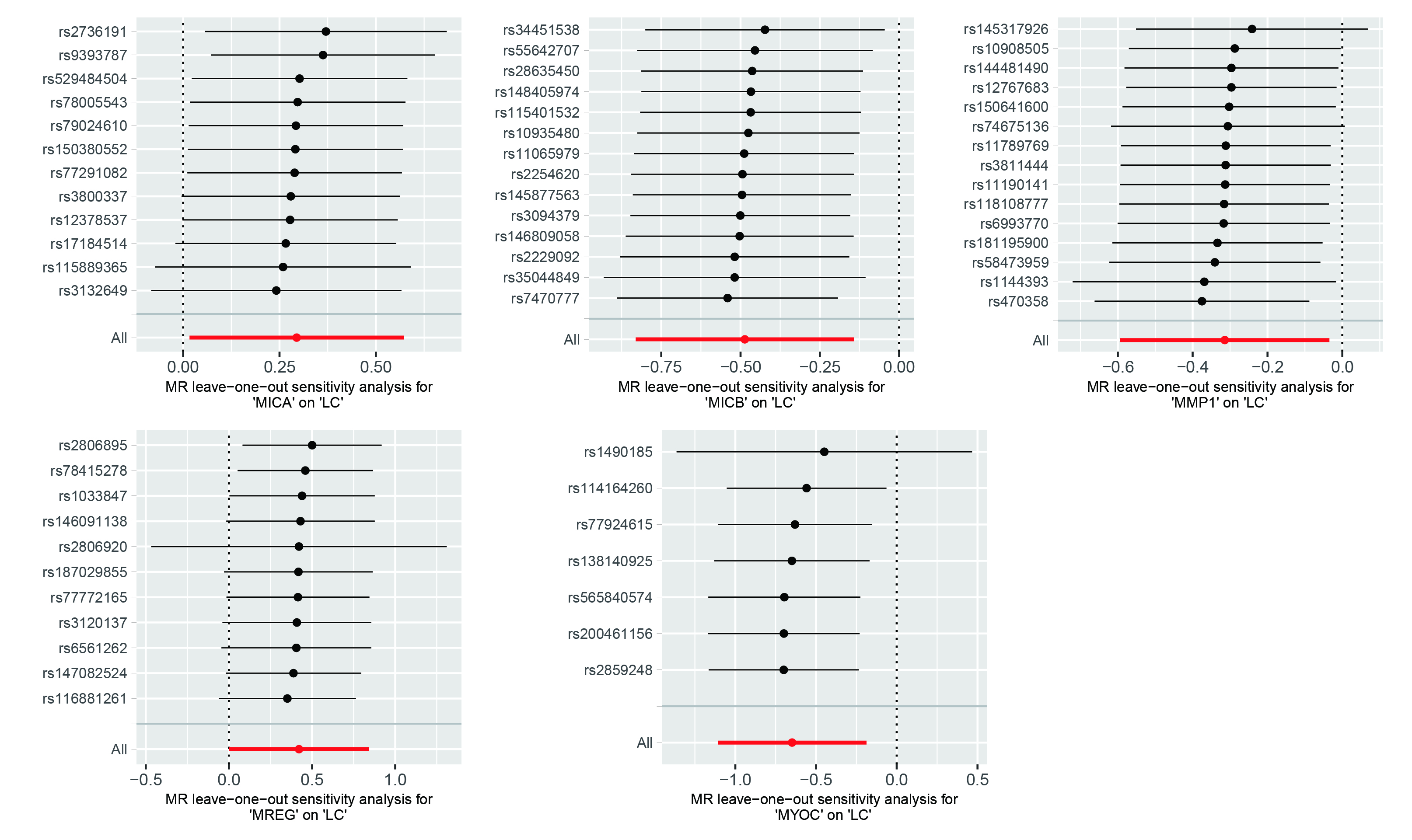

Supplement: Supplementary file 1 [file jpm-14-00262-s001.zip › Supplementary Figure S2.jpg]
